# A nationwide analysis of emergency medical services’ responses during six waves of COVID-19

**DOI:** 10.1177/22799036251396738

**Published:** 2025-11-28

**Authors:** Evan Avraham Alpert, Maximilian P. Nerlander, Bezalel Eliav, Ari M. Lipsky, Ziv Dadon, Roman Sonkin, Eli Jaffe

**Affiliations:** 1 Department of Emergency Medicine, Hadassah Medical Center- Ein Kerem, Jerusalem, Israel; 2 Faculty of Medicine, Hebrew University of Jerusalem, Jerusalem, Israel; 3 Center for Disaster Medicine and Traumatology, Linkoping University, Linkoping, Sweden; 4 Community Division, Magen David Adom, Or-Yehuda, Israel; 5 Department of Emergency Medicine, Shaare Zedek Medical Center, Jerusalem, Israel; 6 Department of Emergency Medicine, HaEmek Medical Center, Afula, Israel; 7 Rappaport Faculty of Medicine, Technion-Israel Institute of Technology, Haifa, Israel; 8 Jesselson Integrated Heart Center, Eisenberg R and D Authority, Shaare Zedek Medical Center, Jerusalem, Israel; 9 Department of Internal Medicine E, Soroka Medical Center, Beer-Sheva, Israel; 10 Department of Emergency Medicine, Faculty of Health Sciences, Ben-Gurion University of the Negev, Beer-Sheva, Israel; 11 Ramat Gan Academic College, Ramat Gan, Israel

**Keywords:** COVID-19, prehospital, big data, infectious diseases, trauma

## Abstract

**Background:**

The COVID-19 pandemic has had significant effects on emergency medical services (EMS). The objective of this study was to investigate how the prehospital response by the Israeli National EMS System (Magen David Adom, MDA) was affected by the first six waves of COVID-19.

**Methods:**

This was a retrospective study using the command-and-control database of MDA from January 1, 2019, through July 31, 2022. EMS responses from each of the six waves of COVID-19 were compared to a historical control period using a 7-day moving average.

**Results:**

A total of 1,242,225 EMS responses were included. During the first wave, there was an increase in daily responses to fever (83.1 vs 40.3; *p* < 0.05) and respiratory symptoms (177.0 vs 151.7; *p* < 0.05), but a decrease for major trauma (78.3 vs 100.4; *p* < 0.05) and motor vehicle accidents (MVA) (44.4 vs 104.4; *p* < 0.05). A similar trend was demonstrated during the second wave. In the third wave, there were no significant differences in responses to respiratory complaints, cardiac complaints, or major trauma. During the subsequent waves, there were significant increases for all types of responses compared to the control periods.

**Conclusions:**

During the first two waves of COVID-19, there was an increase in responses for fever and respiratory symptoms and a decrease in responses for major trauma and MVA. In the subsequent waves, a gradual return to the trend of an overall increase in the number of responses over time compared to the control period was observed.

## Background

COVID-19 has been a significant global challenge with over 774 million documented cases worldwide and over 7 million known deaths, as of March 3, 2024.^1^ Emergency medical services (EMS) have played a major role in the response and management of this pandemic.^2^ However, studies have shown conflicting trends regarding the number of EMS responses that occurred during the pandemic as compared to pre-pandemic periods.

National EMS data from the United States showed an overall decrease in activations from March through December 2020.^3^ A study from Western Pennsylvania during the first wave of COVID-19 compared prehospital responses between March 15 to May 15, 2020, to a similar period in 2016-2019. The article portrays a 26.5% decrease in total EMS responses, including scene responses and interfacility transports.^4^

A study from Ankara, Turkey also showed a decreased number of cases during the pandemic period, which decreased even more during the lockdown period.^5^ Denmark, a country closer in size to Israel, experienced a 3.5% decrease in patients transported to the hospital during the first wave.^6^ Other places that showed a decrease in calls were British Columbia, Tokyo, Tuscany, and the Region Friuli Venezia Giulia in Italy.^7–10^

However, other studies depicted a different trend. A narrative review described a global increase in EMS calls during the early COVID-19 pandemic.^11^ EMS in the Lombardy region of Italy showed an increase of 51.5% in calls during March-April 2020 compared to the same 2 months the previous year.^12^ A retrospective study from Saudi Arabia similarly showed an increase of 52.9% in EMS responses during the pandemic.^13^ Also, initial increases in calls were seen in Bangkok, Thailand,^14^ Tehran, Iran,^15^ and New York City.^16^

Magen David Adom (MDA), Israel’s National Emergency Prehospital Medical and Blood Services Organization, has been heavily involved in managing the epidemic from its beginning. Besides patient transport, this has included the initial screening of suspected cases by phone or at the patient’s home, the establishment of mass drive-through testing centers, and involvement in mass vaccination.^17–20^

Israel was also unique in its early and aggressive campaign to vaccinate the population The campaign to vaccinate with two doses of began on December 20, 2020, and within a few weeks, Israel became the country with the highest percentage of vaccinated individuals worldwide.^21–23^ Subsequently, a third vaccination was given starting on July 30, 2021,^24^ and a fourth one starting on January 2, 2022.^25,26^ In November 2022, Israel started administering the fifth vaccine, which incorporated protection against the Omicron strain.

To date, there is a paucity of studies evaluating EMS activity throughout the various waves of COVID-19. The objective of this study is to describe the epidemiology of EMS responses throughout the six waves of COVID-19 in Israel, compared to the corresponding period before the pandemic, and to contextualize this with periods of social restrictions and vaccine administration.

## Methods

This is a retrospective cohort study using routinely collected programmatic data from MDA’s Command and Control database. All patients treated by MDA ambulances in Israel during the period from January 1, 2019, to July 31, 2022, were included. Variables included demographic data and presenting complaints, which were divided into respiratory complaints, fever, neurological complaints, cardiac complaints, motor vehicle accidents (MVA), major trauma, and “other injuries.” An extensive literature review was conducted to identify a definition of a COVID-19 wave; however little consensus was found in the scientific community. Therefore, total case counts from the Israeli Ministry of Health (MoH) open database were used. Due to great variation in the morphology of the different waves, a mathematical definition was developed where the margins of each wave of COVID-19 were defined as 12% of the sum of the peak height of the wave minus the baseline between waves. The beginning of each wave was calculated relative to the baseline before it and the end of the wave was calculated relative to the baseline after it. Other margins below and above 12% were attempted, however it was found that by using the 12% margin, most of the geometric wave could be incorporated, while avoiding incorporating time periods between waves. Throughout the six waves, the Israeli MoH declared a number of social restrictions. The length of each wave, and the periods of social restrictions are detailed in Table 1 below.

**Table 1.** COVID-19 waves and periods of social restrictions.

Wave	Period	Periods of restrictions
Wave 1	March 21, 2020 to May 5, 2020	From mid-March 2020 to the end of April 2020, a strict lockdown was imposed, including a restriction on leaving the house.
Wave 2	July 3, 2020 to October 30, 2020	From May 2020 to early September 2020, restrictions were not as strict, and subject to frequent changes.
Wave 3	November 30, 2020 to March 23, 2021	From the middle of September 2020 until the beginning of November 2020, The period of social restrictions was tightened again with significant restrictions of movement. From the beginning of November 2020 to the end of December 2020, the restrictions were somewhat eased. At the end of December 2020, social restrictions were imposed again due to an increase in cases, and these were then lifted at the beginning of February 2021.
Wave 4	July 26, 2021 to October 20, 2021	Restrictions were renewed at the end of June 2021 and were eased in early October 2021. There were travel restrictions to certain countries in November 2021 which were lifted on January 6, 2022.
Wave 5	January 8, 2022 to April 3, 2022	Restrictions were finally lifted in early 2022.
Wave 6	May 15, 2022 to July 31, 2022	

EMS responses from each of the six waves of COVID-19 were described using a 7-day moving average. EMS responses were then stratified according to presenting complaint for each wave, where the proportion of each presenting complaint in each wave was compared to a pre-COVID reference period in 2019. This accounted for the seasonal variability of presentations by using each wave’s corresponding calendar dates in 2019. Proportions of binominal variables (type of response for each wave and reference period) were compared using chi-square testing. Odds ratios for each response in the respective waves, as compared to the reference periods were calculated using Pearson’s Chi Squared test. *P*-values <0.05 were considered significant. All data were extracted in Microsoft Excel (One Microsoft Way, Redmond, WA, US) and analyzed in JMP 19 (SAS Campus Drive, Cary, NC, US). This study was approved by the Scientific Committee of MDA and the Helsinki Committee of Shaare Zedek Medical Center (study number 0145-21-SZMC) with a waiver of informed consent.

## Results

A total of 1,243,225 EMS responses were included in the study period (with 924,933 occurring pre-COVID and during the waves) of whom 55.9% were male. The median age was 61.1 years with the largest age subgroup of 80–84 years comprising 9% of the total cohort. During the entire time of the study, the category with the largest number of presentations was “other injuries” (29.7%), whereas respiratory cases comprised the largest non-trauma category (16.7%). The category with the smallest number of presentations was fever (6.9%), closely followed by the neurologic category (8.6%) (Table 2).

**Table 2.** Total EMS Responses Pre-COVID and during the waves, *n* = 924,933 (Total EMS Responses during the study period, *n* = 1,243,225).

	Period (*n* (%))							
Response	Pre-COVID	Wave 1	Wave 2	Wave 3	Wave 4	Wave 5	Wave 6	Total
Respiratory	66,827 (16.6%)	8144 (22.4%)	17,893 (16.3%)	21,881 (19.9%)	15,240 (16.9%)	18,473 (19.6%)	11,696 (14.2%)	160,154 (17.3%)
Fever	22,949 (5.7%)	3823 (10.5%)	11,294 (10.3%)	7523 (6.8%)	7223 (8.0%)	5806 (6.2%)	5393 (6.6%)	64,011 (6.9%)
Cardiac	61,539 (15.3%)	5658 (15.6%)	15,533 (14.2%)	17,411 (15.9%)	13,246 (14.7%)	14,081 (14.9%)	12,223 (14.9%)	139,691 (15.1%)
Neurological	36,787 (9.1%)	3064 (8.4%)	8500 (7.8%)	9365 (8.5%)	6885 (7.6%)	8237 (8.7%)	6916 (8.4%)	79,754 (8.6%)
Major Trauma	45,900 (11.4%)	3600 (9.9%)	12,105 (11.1%)	11,800 (10.7%)	9902 (11.0%)	10,181 (10.8%)	9621 (11.7%)	103,109 (11.1%)
MVA	47,768 (11.9%)	2044 (5.6%)	11,464 (10.5%)	11,214 (10.2%)	10,578 (11.7%)	9885 (10.5%)	10,597 (12.9%)	103,550 (11.2%)
Other Injuries	120,780 (30.0%)	10,032 (27.6%)	32,695 (29.9%)	30,634 (27.9%)	27,099 (30.1%)	27,632 (29.3%)	25,792 (31.4%)	274,664 (29.7%)
Total	402,550 (100.0%)	36,365 (100.0%)	109,484 (100.0%)	109,828 (100.0%)	90,173 (100.0%)	94,295 (100.0%)	82,238 (100.0%)	924,933 (100.0%)

### Trends over the six waves

**Figure 1.**
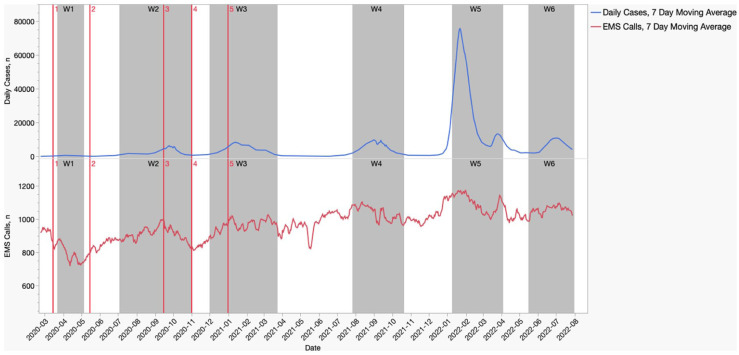
Total EMS Responses and Daily Cases by Date, 7-day Moving Averages (W = wave. Red lines denote start of social restrictions). (*n* = 1,243,225).

Throughout the period of the study, the largest number of daily EMS cases occurred during the fifth wave which also corresponded to the highest number of daily cases (Figure 1). Throughout the first wave, as compared with the reference period, there was an increased relative change in daily responses to respiratory presentations (17% relative increase, *p* < 0.05; OR 1.37, *p* < 0.0001), fever cases (106% relative increase, *p* < 0.05; OR 2.42, *p* < 0.0001) and accompanied by a notable decrease in the total number of daily responses for major trauma (22% relative decrease, *p* < 0.05; OR 0.84, *p* < 0.0001), MVAs (57% relative decrease, *p* < 0.05; OR 0.44, *p* < 0.0001), and other injuries (relative decrease of 17%, *p* < 0.05 OR 0.88, *p* < 0.0001).

A similar trend was partly demonstrated during the second wave showing an increase in respiratory and fever cases. However, the decline in major trauma and MVA cases was less pronounced, and there were no significant differences observed in cardiac cases as well “other injuries” compared to the corresponding period.

**Figure 2.**
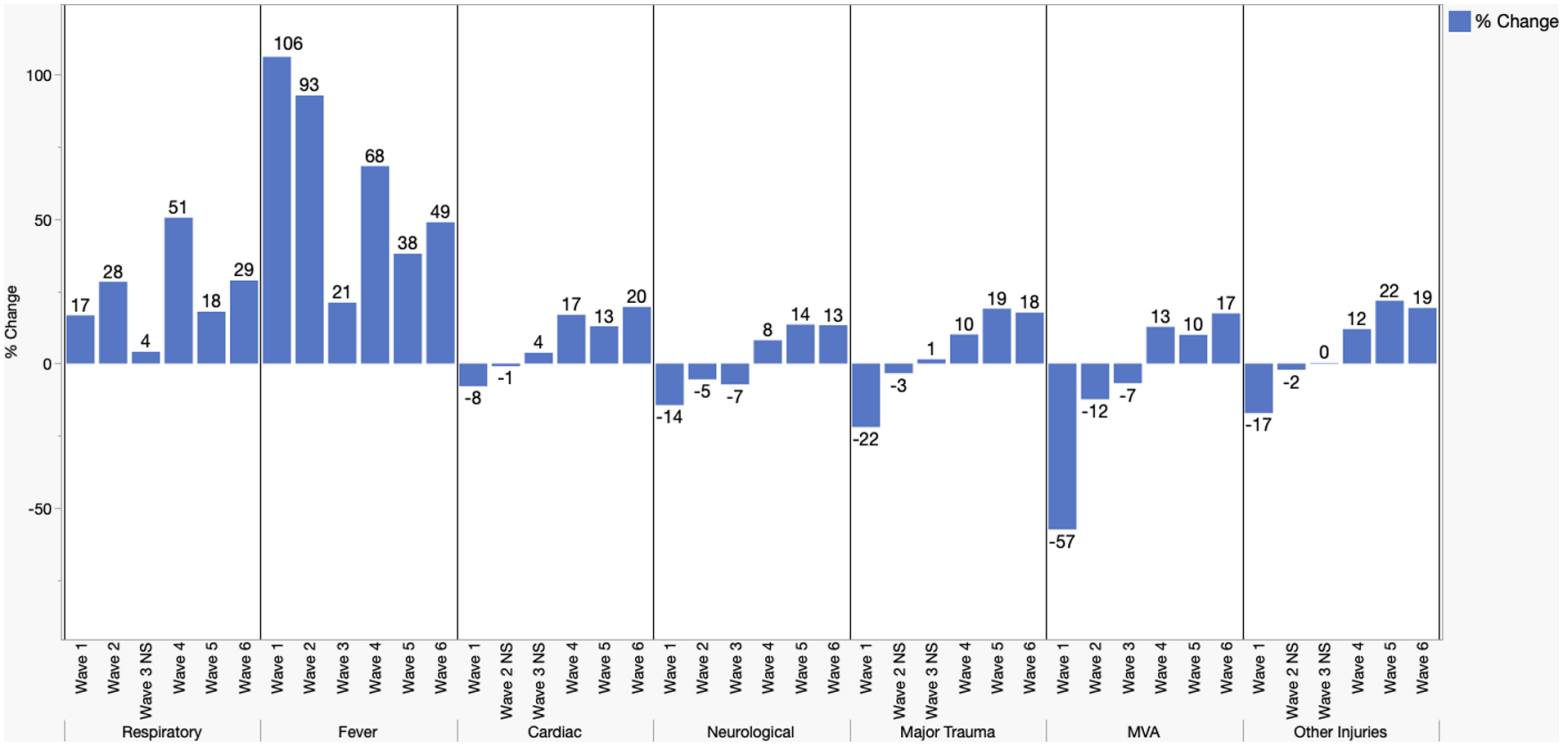
Mean daily responses by COVID-19 wave and response, % change compared to reference period. All changes statistically significant (*p* < 0.05) unless labeled Not Significant (NS). (*n* = 814,479).

Conversely, during the third wave, no statistically significant difference was noted in the number of presentations of respiratory or cardiac cases or responses to major trauma. During the fourth, fifth, and sixth waves, an increase was demonstrated for all types of presentations as compared to the corresponding control period (Figure 2 and Table 3).

**Table 3.** Odds Ratios for Responses Across the Six Waves Compared to Reference Periods. (*n* = 814,479).

	Wave	Odds ratio	95% CI	*p*-value
Respiratory	1	1.37	1.32-1.42	<0.0001
	2	1.26	1.23-1.29	<0.0001
	3	1.03	1.01-1.06	0.0024
	4	1.30	1.26-1.33	<0.0001
	5	1.00	0.98-1.02	0.90
	6	1.07	1.04-1.10	<0.0001
Fever	1	2.42	2.29-2.57	<0.0001
	2	1.92	1.86-1.98	<0.0001
	3	1.21	1.17-1.25	<0.0001
	4	1.43	1.28-1.49	<0.0001
	5	1.18	1.13-1.23	<0.0001
	6	1.25	1.19-1.30	<0.0001
Cardiac	1	1.02	0.98-1.06	0.3617
	2	0.93	0.9-0.95	<0.0001
	3	1.03	1.00-1.05	0.0200
	4	0.96	0.94-1.00	0.0061
	5	0.95	0.92-0.97	<0.0001
	6	0.99	0.96-1.01	0.3018
Neurological	1	0.94	0.89-0.99	0.0141
	2	0.89	0.86-0.91	<0.0001
	3	0.91	0.88-0.93	<0.0001
	4	0.89	0.85-0.92	<0.0001
	5	0.96	0.93-0.99	0.0102
	6	0.93	0.90-0.96	<0.0001
Major trauma	1	0.84	0.81-0.88	<0.0001
	2	0.90	0.88-0.93	<0.0001
	3	1.00	0.98-1.03	0.9009
	4	0.90	0.87-0.93	<0.0001
	5	1.01	0.98-1.04	0.5977
	6	0.97	0.94-1.00	0.0349
MVA	1	0.44	0.41-0.46	<0.0001
	2	0.81	0.79-0.83	<0.0001
	3	0.91	0.89-0.94	<0.0001
	4	0.93	0.90-0.95	<0.0001
	5	0.92	0.90-0.95	<0.0001
	6	0.96	0.94-0.99	0.0168
Other injuries	1	0.88	0.85-0.91	<0.0001
	2	0.89	0.88-0.91	<0.0001
	3	0.98	0.97-1.00	0.0982
	4	0.90	0.88-0.92	<0.0001
	5	1.04	1.02-1.07	<0.0001
	6	0.98	0.96-1.00	0.0385

## Discussion

This study investigated all-cause EMS responses at a national level during six waves of the COVID-19 pandemic in Israel. Statistically significant differences are evident throughout all categories of EMS presentations compared to the corresponding period before COVID-19, throughout all six waves. These trends were likely influenced by the lockdowns detailed above as well as the administration of vaccinations and the subsequent reassurance and relief among the public.

### Overall data

During the first wave, a significant decrease was observed in the total number of calls, presumably driven by a decline in all non-COVID-19 related categories. These findings are similar to those observed in other studies of the first wave.^3–5,7–10,27,28^ It should be noted that conflicting results were observed by different studies showing an increase in the number of EMS cases during the first wave,^11–16,29^ or that the total volume remained consistently stable.^30,31^ The increase in the number of EMS calls, in Thailand can be explained by the accessibility of the emergency services, the fact that it is provided free of charge by the government, and that according to public opinion, it is a relatively safe way to access the public health system.^14^ However, for many of the other studies that reflect conflicting results during their country’s initial COVID-19 EMS response, there is no clear distinction among the different geographic locations. This finding precludes the formulation of decisive conclusions regarding possible distinctions that may explain differing trends, including the availability and robustness of outpatient clinics as well as the nature of the EMS system and the strictness of the lockdowns leading to different health-seeking behaviors. Of note, some of the discrepancies may also be attributed to a different methodology according to which they did not report calls that weren’t followed by transport to the hospital, while others did.^32^ However, the fifth wave had the highest number of daily cases which was probably due to the highly transmissible Omicron variant.

### Respiratory and fever-related presentations

Presentations secondary to respiratory complaints and infectious-related symptoms were shown in the current study as well as many others to increase during the beginning of the pandemic and the first waves^11–16,29–32^ even by those showing an overall decrease in the number of presentations.^4,5,7,9^ Interestingly, the number of COVID-19 patients in Israel during the first wave was relatively low as can be seen in Figure 1, and therefore the increase in EMS responses for fever and respiratory symptoms shown in the present study cannot be attributed solely to the disease burden of COVID-19.^21^ It can be assumed that the high public awareness had an impact on health-seeking behavior leading concerned people with non-COVID-19 acute respiratory or infectious illness to ask for assessment in the hospital settings. During the first year of COVID-19 there was a decrease for in person visits to primary care clinics during the beginning of the pandemic. Although there was an increase in virtual visits, such as via phone conversations or telemedicine, the desire to see a clinician in real time may have resulted in an increased utilization of EMS and ED services.^33,34^

### Cardiac-related presentations

Presentations categorized as cardiac-related cases encompassed a broad spectrum of symptoms, including chest pain, acute coronary syndrome, arrhythmias, cardiogenic shock, and pulmonary edema. Unfortunately, the data regarding resuscitations or out-of-hospital cardiac arrest were not specifically collected under this category. The present study shows a decrease in the number of cardiac cases only during the first wave, after which it plateaued for the second and third waves, and then increased for the remaining three waves. The declining trend shown during the beginning of the pandemic closely mirrored the trajectory seen in the general cases, however, a quicker normalization was observed. Most studies reported similar findings, showing a decline in the general cardiac-related cases during the beginning of the pandemic^11,15,30,35^ though some showed an increase^16^ or no-change^10^ in these cases. The differing results may stem from inconsistent diagnoses included under the cardiac category. Also, it is possible that different trends within the sub-diagnoses included in this broad cardiac category have led to this apparent discrepancy. For example, ST-elevation myocardial infarction (STEMI) presentations to EMS were shown to decrease,^7,9,30^ results that may result from different patient behavior, including fear and confusion leading to a change in STEMI presentation and care.^30,36^ During the second wave, similar changes to the first wave can be seen, but on a smaller scale. The decrease in the intensity of the changes can be attributed to the looser social restrictions during this period. The third wave presents no significant change compared to the comparison period. These findings can be attributed to the better adaptation of the health system to the treatment of patients within the community and a decrease in the number of evacuations to the hospital. Another possible contributing factor to these findings is the distribution of vaccines in Israel during the third which resulted in a milder presentation of COVID-19, as demonstrated in other studies.^24,37^

During the fourth through sixth waves, a general increase in the total number of cardiology cases was observed. These findings can be explained mainly by the vaccination effect, which ended the third wave and was a decisive factor in dealing with the pandemic later on.

### Neurology-related presentations

Presentations classified as neurologic cases (incorporating mainly suspected cerebrovascular accidents (CVA) or transient ischemic attacks) decreased during the first three waves, with the return to normality from the fourth wave - a trend that resembles the one shown in the total number of cases. Conflicting results were shown by different studies regarding CVA-related EMS presentations, with studies demonstrating either an increase^38^ decrease^7,10,11^ or no significant change^39,40^ in cases. Some of the discrepancy may be attributed to the notion that in some areas COVID-19 appears to have affected the stroke chain of survival by hindering entry into EDs with stroke centers.^41^

### Trauma-related presentations

A consistent trend that is demonstrated by most studies describing the topic, though not shared by all^14^ is a decrease in the volume of transports for major trauma, motor vehicle accidents, and other injuries during the period when lockdowns were applied, a finding also shown in the present study.^3–5,7–10,13,15,16,28,42^ These findings were likely driven by a significant reduction in vehicle activity, a significant decrease in workplace activity, and shutdown of most of the education systems, where traumatic injuries frequently occur.^43,44^ A study from Ireland showed that the majority of trauma during the pandemic appeared to be home-based with significant reductions apparent in work- and sport-related injuries.^45^ A study carried out at a children’s ED in Portugal showed a decrease in sports injuries and injuries at school, but an increase in home injuries such as burns and dog bites.^46^

## Limitations

Although this study is unique in that it is a large national database that covers six waves of COVID-19, there are several limitations. The very nature of EMS is such that they work on presumed diagnoses in the field without knowing the final hospital diagnosis. Therefore, The focus of this prehospital epidemiologic study is on the presumptive diagnosis of EMS during six waves of COVID-19., the respiratory-related EMS category is therefore broad and may include diseases such as congestive heart failure as well as shortness of breath secondary to COVID-19. The cardiac-related presentations are also very broad and may include musculoskeletal chest pain as well as a STEMI. Another issue is that the study does not look at outcomes. The patients’ complaints of fever or respiratory symptoms may not necessarily be translated into positivity for COVID-19.

## Conclusion

Lessons from this analysis of a large pre-hospital database may help EMS systems better prepare for future pandemics. For example, during the first two waves before a massive vaccine program began, there was an increase in respiratory cases. Pre-hospital systems at that point need to invest in equipment and training related to respiratory symptoms. This may include N-95 masks and protective gear. Training and education should emphasize protection from respiratory borne pathogens and how to best manage the dyspneic patient. As the public becomes vaccinated these investments and precautions can be adjusted accordingly.
